# Acute and Subchronic Oral Toxicity of Fermented Green Tea with Aquilariae Lignum in Rodents

**DOI:** 10.1155/2019/8721858

**Published:** 2019-09-10

**Authors:** Sol Lee, Phil Hyun Song, Young Joon Lee, Sae-Kwang Ku, Chang-Hyun Song

**Affiliations:** ^1^Department of Anatomy and Histology, College of Korean Medicine, Daegu Haany University, Gyeongsan 38610, Republic of Korea; ^2^Department of Urology, College of Medicine, Yeungnam University, Daegu 42415, Republic of Korea; ^3^Department of Preventive Medicine, College of Korean Medicine, Daegu Haany University, Gyeongsan 38610, Republic of Korea

## Abstract

Green tea is generally considered safe, but there have been concerns regarding side effects relating to the main component, catechins, especially hepatotoxicities. We have previously shown beneficial effects of fermented green tea with Aquilariae Lignum (fGT) via an oral route in diabetic and obese models. Thus, the toxicological safety of fGT was assessed at limited oral doses for a rodent. Mice or rats of both genders were orally administered distilled water as a control and fGT at 2.0, 1.0, and 0.5 g/kg. There were no mortalities or gross abnormalities in the fGT groups for 2 weeks following the single oral dose in mice. No fGT-relevant abnormalities were found in postmortem and histopathological examinations, suggesting LD_50_ of fGT at more than 2.0 g/kg with no specific target organs. There were also no fGT-relevant mortalities or abnormal signs in the repeated oral dose for 13 weeks in rats. In the fGT groups, no body weight changes or daily metabolic changes were found, and hematological and serum biochemical ranges were normal. The postmortem and histopathological examinations revealed few fGT-related abnormalities in most of the organs including the liver, although slight lymphoid cell hyperplasia in the lymph node was observed in a few rats with fGT at 2.0 g/kg. This may be secondary to increased immune response to the highest dose because there were no histopathological lesions or organ weight changes. It suggests nontoxic safety of fGT at up to 2.0 g/kg, which provides useful information for clinical use.

## 1. Introduction

Tea prepared from dried leaves of *Camellia sinensis* L. is the second most consumed beverage after water in the world [1]. Tea has been traditionally used as a medicinal plant in Asian countries including India, China, and Japan [2], and it is believed to have potential effects on prevention or treatment of many diseases, including cancer, cardiovascular disease, and neurodegenerative disease [3]. The relevant active components include polyphenols (catechins and flavonoids), alkaloids (e.g., caffeine, theophylline, and theobromine), amino acids, and vitamins [4, 5]. In particular, the polyphenols are primarily responsible for the beneficial effects of tea, and the flavonoids have antioxidant, anti-inflammatory, antiallergic, and antimicrobial effects [3]. Tea is divided into unfermented green tea, partially fermented oolong tea, and fermented black tea, according to the treatment of dried leaves [6]. The fermentation of tea converses the main ingredients by activated oxidation [7]; black tea contains less polyphenols but more flavonoids and caffeine, compared with green tea. Different chemical compositions depending on various tea fermentation and manufacturing processes affect their bioactivities [8–10], which need careful safety assessment.

The annual increase of tea consumption is currently driven by its use in various health beverages or food supplements. Green tea and its main component, catechins, are generally considered safe. However, concerns have been raised regarding side effects of epigallocatechin gallate (EGCG), which accounts for more than half of total catechins and is one of the most biologically active green tea ingredients, on hepatotoxicity and hemorrhagic lesions in the gastrointestinal tracts [11, 12]. In addition, high doses of green tea polyphenols have been reported to have harmful effects on the reproductive systems [13]. The side effects seem to occur more notably when tea is taken in a fasting state as a supplement tablet. However, the levels of total catechins and EGCG decrease with the degree of fermentation [14], in the order of green tea, oolong tea, and black tea. Furthermore, fermented herbs have been highlighted as a new source of medicinal ingredients because fermentation enhances the pharmacological efficacy of the parent herbs through biotransformation or probiotic effects [15–17].

Aquilariae Lignum, the stem part of *Aquilaria agallocha* Roxb. (Thymelaeaceae), has been used as an aroma-therapeutic reagent, or in traditional medicine in Asia to treat cough, acroparalysis, courap, and asthma [18, 19]. It contains benzylacetone, p-methoxybenzylacetone, hydrocinnamic acid, agarospirol, agarofuran, and dihydroagarofuran [20], which are shown to have sedative, analgesic, immune-modulatory, antioxidant, and anti-inflammatory properties [21–25]. Furthermore, Aquilariae Lignum contributes to the characteristic flavor of various teas, making them popular worldwide, aside from their medicinal properties. Our previous studies have shown that aqueous extracts of green tea fermented with Aquilariae Lignum (fGT) ameliorate diabetes and its related complications by inhibiting hyperlipidemia, hepatopathy, nephropathy, and obesity in db/db and high-fat diet animal models [16, 26]. The favorable effects of fGT are greater than those of green tea. Nevertheless, there have been no toxicological profiles on intake of Aquilariae Lignum or fGT. We examined the acute and subchronic oral toxicities of fGT at limited dosages in rodents, to clarify its safe threshold intake level.

## 2. Materials and Methods

### 2.1. fGT Preparation

The fGT was provided by ChuiWoon HyangDang (Seongju, Korea); it was the same as that used in our previous study [16]. In brief, a mixture of dried green tea leaves and Aquilariae Lignum powder (49 : 1, g/g) was wet-fermented for 12 h at 60°C and dried for a week at 15°C. It was steamed for 30 sec at 100°C and further dried for 3 days at 15°C. Then, it was boiled for 6 h and lyophilized. While the dried green tea contained 8.24% catechins and 6.68% caffeine as main ingredients, the fGT contained 1.53% catechins and 7.79% caffeine.

### 2.2. Animals

Six-week-old CrljOri: CD1 (ICR) mice and CrljCD (SD) rats were purchased from Orient Bio, Inc. (Seongnam, Korea). Five animals were allocated per polycarbonate cage and housed in a temperature (20–25°C) and humidity (40–45%) controlled room with a light/dark cycle of 12/12 h. Feed and water were supplied free to access. After acclimatization for a week, the mice and rats were used for examining single and repeated oral dose toxicity of fGT, respectively. The animals were fasted overnight before the initial administration and euthanasia using CO_2_ gas, to avoid diet effects. All animal experiments were conducted according to international regulations on the use and welfare of laboratory animals and approved by the institutional animal care and use committee of Daegu Haany University (Gyeongsan, Korea, approval numbers DHU2015-037 and DHU2015-039 for the single and repeated oral toxicity studies, respectively).

### 2.3. Experimental Design

For a single oral dose of fGT, a total of 40 male and female ICR mice were assigned to four groups of both genders (*n* = 5 per group each), with a similar variance of body weights. The mice received a single oral administration using a gastric gavage as follows: distilled water as a vehicle control (Control) and fGT at 2.0, 1.0, and 0.5 g/kg (fGT2.0, fGT1.0, and fGT0.5, respectively) in a volume of 20 ml/kg. The repeated oral dose toxicity was examined in a total of 40 male and female SD rats at the same experimental design with the single oral dose. Oral administration was performed once a day for 13 weeks in a volume of 10 ml/kg. The dosage of fGT was determined at the maximum dose for a rodent, based on guidelines of OECD [27] and Korea Food and Drug Administration [28]. Abnormal clinical signs were recorded by functional observational battery tests twice a day [29], and body weights were measured.

### 2.4. Food and Water Consumption and Fecal and Urine Excretion

A rat was housed in each individual metabolic cage and supplied a diet of 150 g and 250 ml of water. After housing for 24 h, the remaining diets and water were measured for daily consumption, and fecal weight and urine volume were measured for the excretion amounts.

### 2.5. Hematological and Serum Biochemical Analyses

Rat blood (more than 6 ml) was drawn from the inferior vena cava under anesthesia with 2-3% isoflurane in a mixture of 70% N_2_O and 28.5% O_2_. A portion of the samples were collected into CBC bottles containing EDTA-2K at 1.8 mg/ml for hematology, and other samples were centrifuged at 1700 ×*g* for 10 min at 4°C for serum biochemistry. 13 items of hematological abnormalities were examined, including numbers of total leukocytes and the differential numbers (neutrophils, eosinophils, basophils, lymphocytes, and monocytes), erythrocytes, platelets, hemoglobin concentration, hematocrit, mean corpuscular (MC) volume, MC hemoglobin (MCH), and MCH concentration. Twenty items of serum biochemical abnormalities were examined, including alkaline phosphatase (ALP), aspartate aminotransferase (ASP), alanine aminotransferase (ALT), lactate dehydrogenase (LDH), blood urea nitrogen, total bilirubin, glucose, cholesterol, triglycerides, creatine phosphokinase, creatinine, total protein, albumin, globulin, albumin/globulin ratio, and inorganic phosphorus, calcium, sodium, potassium, and chloride. The hematological and serum biochemical examinations were performed in the veterinary hospital of the College of Veterinary Medicine of Kyungpook National University (Daegu, Korea), using Cell-Dyn 3700 (Abbott Laboratories, Abbott Park, IL, USA) and Dri-Chem NX500i (Fuji Medical Systems Co., Ltd., Tokyo, Japan), respectively.

### 2.6. Postmortem Examination (Necropsy)

Postmortem abnormal changes were examined mainly in 26 specific organs including the lung, heart, spleen, thymus, submandibular lymph nodes and salivary gland, gastrointestinal (GI) tracts (esophagus, stomach, duodenum, jejunum, ileum, cecum, colon, and rectum), liver, pancreas, kidney, adrenal gland, urinary bladder, prostate, testis, epididymis, ovary, uterus, brain, and skin. The scores were assessed as +1, +2, and +3 for slight, moderate, and severe changes, respectively, by a pathologist blinded to the group [30]. The organ samples were weighed and expressed as a percentage of the body weight.

### 2.7. Histopathological Examination

Organ samples were fixed in 10% neutral buffered formalin. They were paraffin-embedded and sectioned at a thickness of 3-4 *μ*m. The sections were stained with hematoxylin and eosin (H&E), and any abnormal findings were recorded using a computer-based image analysis program (i-Solution FL ver. 9.1; IMT i-Solution Inc., Vancouver, BC, Canada). The scores were assessed as +1, +2, and +3 for slight, moderate, and severe changes, respectively, by a pathologist blinded to the group [30, 31].

### 2.8. Statistical Analysis

Values were expressed as mean ± standard deviation (SD). Homogeneity of variance was examined using the Levene test. In case of no significance in the test, data were analyzed by a one-way analysis of variance (ANOVA) test, followed by the Scheffe post hoc test. In case of significance, nonparametric comparison was conducted by the Kruskal–Wallis *H* test, followed by the Mann–Whitney (MW) *U* post hoc test. The kinetic data from body weights and daily metabolic changes were examined by two-way ANOVA with main factors of the group and time points measured, and the time point was treated as a repeated measure. The result was regarded as significant when the *P* value was less than 0.05.

## 3. Results

### 3.1. Acute Oral Toxicity of fGT

#### 3.1.1. Mortalities and Gross Abnormalities after the Single Oral Dose

Acute toxicity of fGT was examined in both genders of mice for two weeks after the single oral dose. There were no mortalities in all groups of both genders. No clinical abnormalities were found in all groups. The body weights were measured on a day before treatment and days 0, 1, 2, 7, 13, and 14 after treatment. The weight changes were not significantly different among groups ([Supplementary-material supplementary-material-1]).

#### 3.1.2. Postmortem Abnormalities after the Single Oral Dose

There were few abnormalities in the specific organs of all groups of both genders, except some abnormal changes in five organs: the lymph nodes, spleen, thymus, lung, and uterus (Table 1). The changes were hypertrophy in the lymph node, atrophy in the spleen and thymus, congestion in the lung, and edema in the uterus, and they were observed in just a few mice in all groups, including the control group. However, the number of mice showing abnormalities was the same or fewer in fGT2.0, the highest dose group, compared with control group. Most abnormalities were also not more frequent in fGT1.0 and fGT0.5 than in the control group, although one or two more mice showed the lymph node hypertrophy and the uterine edema in fGT1.0 and fGT0.5, respectively. The absolute and relative weights of the 14 organs, including the lymph node and uterus, were not different in the fGT groups of both genders compared with the control group (Tables [Supplementary-material supplementary-material-1] and 2).

#### 3.1.3. Histopathological Abnormalities after the Single Oral Dose

The histopathological examination showed no abnormalities in the specific organs of all groups except slight changes in four organs: the lymph nodes, spleen, liver, and lung. Consistent with the results from the postmortem examination, lymphoid cell (LC) hyperplasia in the lymph node, the decreased number of LCs in the splenic white pulp, and congestion in the lung were found in a few mice of all groups, but the thymus and uterus were not abnormal in any group ([Supplementary-material supplementary-material-1]). Histopathological abnormalities were observed in the same or fewer mice of fGT2.0, when compared with mice of the control group (Table 3). Although one more mouse of the fGT1.0 group showed abnormal changes in the lymph node, the other abnormalities were not more in fGT1.0 and fGT0.5 than in the control group. Slight inflammatory cell infiltration in the liver was observed in one mouse of the female fGT1.0 group only. Because there were no more dose-dependent abnormalities in the fGT groups of both genders than in the control group, the subchronic oral toxicity study was performed.

### 3.2. Subchronic Oral Toxicity of fGT

#### 3.2.1. Mortalities and Gross Abnormalities after the Repeated Oral Dose for 13 Weeks

**Figure 1:**
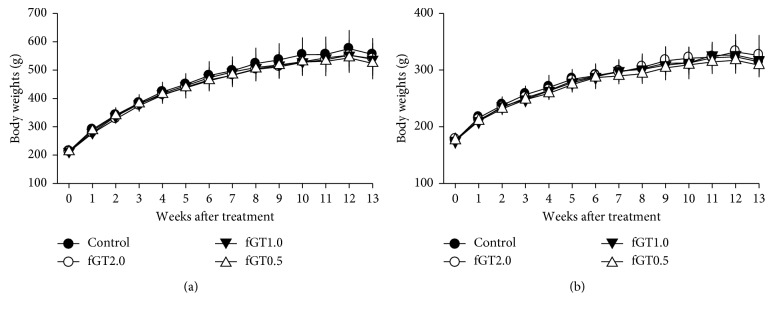
Body weight changes after a repeated oral dose of fGT for 13 weeks. Body weights of male (a) and female (b) rats are expressed as mean ± standard deviation (SD) (*n* = 5 per group each).

There were no mortalities or abnormal signs in any group. The kinetic changes of body weight were also not different in the fGT groups of both genders as compared with the control group (Figure 1).

#### 3.2.2. Daily Metabolic Changes after the Repeated Oral Dose for 13 Weeks

**Figure 2:**
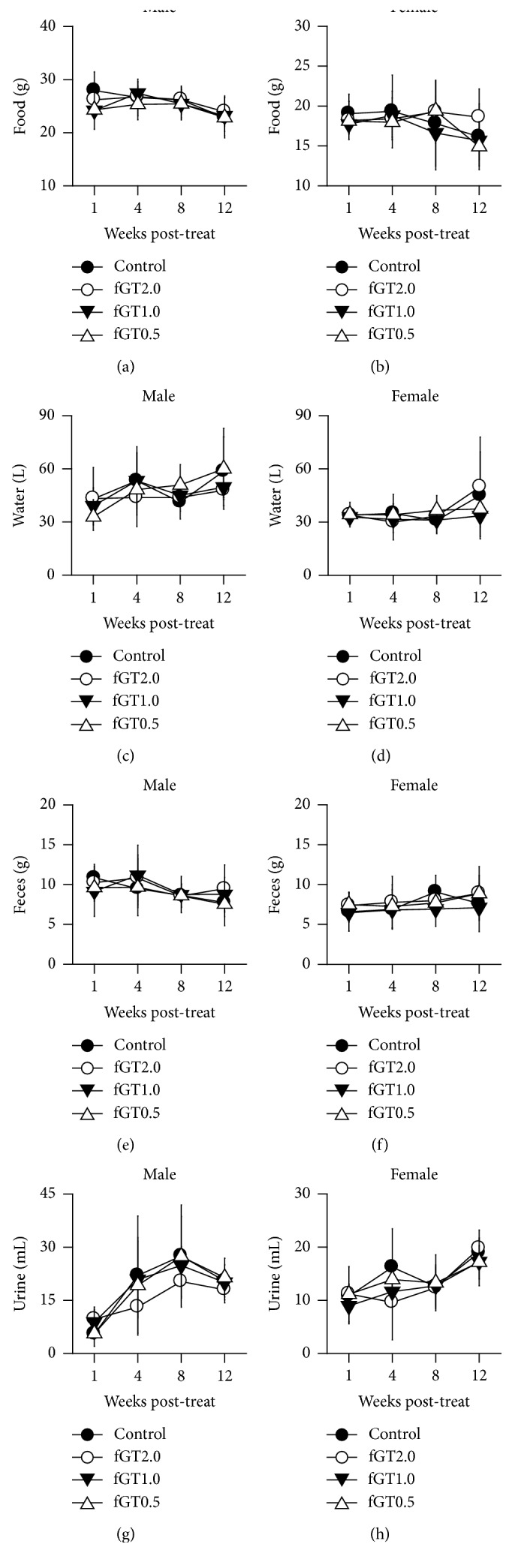
Metabolic changes after a repeated oral dose of fGT for 13 weeks. Male and female rats (*n* = 5 per group each) were orally administered distilled water (Control) and fGT. The food (a, b) and water (c, d) consumption and the fecal (e, f) and urine (g, h) excretion were assessed on the time points indicated. Values are expressed as mean ± SD.

Daily metabolic changes were examined on weeks 1, 4, 8, and 12 after treatment. There were no significant differences in food and water consumption or in fecal and urine excretion in the fGT groups of both genders, compared with those of the control group (Figure 2).

#### 3.2.3. Hematology and Serum Biochemistry after the Repeated Oral Dose for 13 Weeks

There were no significant differences in 13 hematological items in the fGT groups of both genders compared with the control group, except basophils in the male fGT0.5 group (Table 4). The basophils declined by 28% in fGT0.5 compared with the control group (*P* < 0.05). Furthermore, no meaningful changes were found in 20 serum biochemical items in the fGT groups of both genders compared with the control group, except the sodium content in the female fGT1.0 group and total bilirubin and chloride contents in the male fGT0.5 group (Table 5). Compared to the control group, the female fGT1.0 group showed a significant decrease by 1.7% in sodium and the male fGT0.5 group showed a decrease by 50% in total bilirubin and an increase by 4.2% in chloride (*P* < 0.05). Although these exceptional significances were found, the results were still in normal hematological and serum biochemical ranges [32].

#### 3.2.4. Postmortem Abnormalities after the Repeated Oral Dose for 13 Weeks

There were slight abnormal changes showing hypertrophy in the lymph node and congestion in the lung in all groups but few abnormalities in the other specific organs (Table 6). While the lung congestion in the fGT groups of both genders was observed to be similar to that in the control group, the lymph node hypertrophy was observed in two more mice of the fGT2.0 group as compared to the control group. The lymph node hypertrophy was not greater in the fGT1.0 and fGT0.5 groups than in the control group. There were no significant differences in absolute and relative weights of the specific organs in the fGT groups of both genders, compared with the control group (Tables [Supplementary-material supplementary-material-1] and 7).

#### 3.2.5. Histopathological Abnormalities after the Repeated Oral Dose for 13 Weeks

There were slight abnormal changes in eight organs of all groups but few abnormalities in other specific organs. The abnormal changes were LC hyperplasia in the lymph node, the decreased number of LCs in the spleen, congestion in the thymus, lung, and adrenal gland, inflammatory cell infiltration in the liver, and tubular atrophy in the prostate ([Supplementary-material supplementary-material-1]). Abnormalities in the thymus, liver, lung, adrenal gland, and prostate were observed in an equal number of mice or fewer mice of the fGT groups compared with the control group (Table 8). The abnormal changes in the spleen in the fGT groups were also similar to those in the control group, although there was one more mouse in the female fGT1.0 group. Slight changes of pyloric cysts were observed in one mouse of the female fGT1.0 group only. LC hyperplasia in the lymph node was detected in two more mice of the fGT2.0 group compared with the control group, while in the fGT1.0 and fGT0.5 groups, it was similar to that in the control group.

## 4. Discussion

The fermentation process of green tea with Aquilariae Lignum for fGT results in a different chemical composition [16, 26], which requires a safety assessment for further clinical use. The acute and subchronic oral toxicity studies showed nontoxic safety of fGT at up to 2 g/kg/day.

The single oral toxicity study showed no fGT-related mortalities or gross abnormalities including abnormal signs and body weight changes. Slight changes in the lymph node, uterus, and liver were observed in one or two more female mice of the fGT1.0 and fGT0.5 groups only than in the control group, but other changes in the fGT groups were similar to those in the control group. In particular, the postmortem and histopathological analyses revealed few abnormal changes in the fGT2.0 group, the highest dose group. Similarly, there were no acute oral toxicities of fermentation-processed black tea at up to 2 g/kg in Swiss albino mice [33] and no subacute oral toxicities of green tea extracts at up to 2.5 g/kg for 28 days in ICR mice [34]. Acute oral toxicity of tea seems to have a positive relation with EGCG contents; the approximate lethal dose (LD_50_) is between 186.8 and 1,868 mg/kg in rats [35], and severe hepatotoxicity is at 1,500 mg/kg in mice [36]. Here, the administered catechins (1.53% in fGT) including EGCG can be calculated as about 30.6 mg/kg in the fGT2.0 group, which may be a safe dose within the LD_50_ range. Although a few changes were found in the fGT1.0 or fGT0.5 group, the results were regarded as just sporadic changes rather than the fGT-specific target-organ injuries for the following reasons: the abnormalities were detected in the control group as well and dose independent in the fGT group and were not accompanied by abnormal specific organ weights or the relevant histopathological lesions. This suggests LD_50_ of fGT at more than 2 g/kg with no specific target organs in mice of both genders.

No subchronic oral toxicities of black tea at up to 250 mg/kg have been reported; no abnormalities of body weight, food consumption, hematology and serum biochemistry, organ weight, and histopathology were reported [33]. There were herein no mortalities or abnormal signs in the repeated oral dose toxicity of fGT at the limited dosage for rodents. No body weight changes were found in the fGT groups compared with the control group, although the same fGT has shown significant weight loss in db/db and high-fat diet mice via an oral route at the lower dosages of 0.4, 0.2, and 0.1 g/kg [16, 26]. Daily metabolic changes are an important factor affecting body weight, but the fGT groups showed no changes in food and water consumption or fecal and urinary excretion. It may be related to an interaction of the ingredients of fGT with lipids, considering that tea polyphenols increase fecal lipids [37], and coadministration of tea catechins with butter increases fecal excretion [38]. The hematological and serum biochemical items of the fGT groups were in normal ranges, although some significances were found in the lower-dose groups of fGT compared with the control group [32]. The postmortem and histopathological examinations showed some slight sporadic changes in the spleen and the gastric pylorus of the female fGT1.0 group only. However, LC hyperplasia in the lymph node was nonsignificant in a few more mice in the fGT2.0 group than in the control group. Conversely, toxic effects of tea have shown atrophic changes in the mesenteric and mandibular lymph nodes with numerous apoptosis [39]. There were no differences in organ weights including the lymph node and no relevant lesions in the fGT2.0 group. It has been reported that fGT has stronger antioxidant properties than the parent green tea [16] and that black tea improves immune response [40]. In this context, the changes in the lymph node were considered to be secondary effects by increased immune response to the highest dose of fGT.

Subchronic oral doses of green tea extracts at 1.0 g/kg have shown treatment-related mortalities in B6C3F1 mice but not in F344/NTac rats [39]. The animal deaths were related to liver necrosis, and the no adverse effect level (NOAEL) of green tea extracts in the liver of both mice and rats was 500 mg/kg/day. Other subchronic oral toxicity studies have shown the NOAEL of green tea catechins was 764 mg/kg/day for male rats and 820 mg/kg/day for female rats [41] and of EGCG was 500 mg/kg/day in rats [42]. Here, there were no fGT-relevant hepatotoxicities. Only inflammatory cell infiltration in the liver was observed similarly in all groups including the control group. The fGT groups showed no abnormalities in liver weights or in hematological and serum biochemical items involved in the inflammation (i.e., leukocytes) or liver injuries (i.e., ALP, AST, ALT, LDH, total bilirubin, albumin, globulin, and albumin/globulin ratio). Rather, reduction of basophils was observed in the male fGT0.5 group. In connection with previous studies, the reduced content of catechins via additional oxidization in the fGT may have little hepatotoxicities [43], or enhanced antioxidant activities may contribute to hepatoprotective effects [16, 26, 44, 45]. Green tea tends to be less fragrant and more bitter than the fermented teas, although it is popular as a health drink. Lower levels of catechins and higher levels of theanines have been reported to improve the tea taste and aroma [46]. Furthermore, Aquilariae Lignum as an aroma-therapeutic reagent may reduce the bitter and astringent taste of green tea and enhance its characteristic fragrance. Current toxicological studies provide useful information for the clinical use of fGT as a medicinal herb or a health beverage.

## Figures and Tables

**Table 1:** Postmortem abnormalities after a single oral dose of fGT.

	Control (male, female)	fGT (male, female)		
		2.0 g/kg	1.0 g/kg	0.5 g/kg
LN: hypertrophy +1	1/10 (1/5, 0/5)	1/10 (1/5, 0/5)	2/10 (1/5, 1/5)	1/10 (1/5, 0/5)
Spleen: atrophy +1	2/10 (1/5, 1/5)	1/10 (1/5, 0/5)	1/10 (0/5, 1/5)	2/10 (1/5, 1/5)
Thymus: atrophy +1	2/10 (1/5, 1/5)	0/10 (0/5, 0/5)	0/10 (0/5, 0/5)	2/10 (1/5, 1/5)
Lung: congestion +1	3/10 (2/5, 1/5)	2/10 (1/5, 1/5)	2/10 (1/5, 1/5)	2/10 (1/5, 1/5)
Uterus				
Edema +1	1/5 (—, 1/5)	1/5 (—, 1/5)	0/5 (—, 0/5)	3/5 (—, 3/5)
Edema +2	1/5 (—, 1/5)	1/5 (—, 1/5)	1/5 (—, 1/5)	0/5 (—, 0/5)

Mice (*n* = 5 per group each) were orally administered distilled water as a vehicle control (Control) or aqueous extracts of fermented green tea with Aquilariae Lignum (fGT). Abnormal changes are scored as +1 and +2 for slight and moderate changes, respectively. Mice showing the abnormalities are listed as a number per total number. LN = lymph node.

**Table 2:** Relative organ weights after a single oral dose of fGT.

	Control (male/female)	fGT (male/female)		
		2.0 g/kg	1.0 g/kg	0.5 g/kg
Brain	1.45 ± 0.05/1.90 ± 0.16	1.45 ± 0.07/1.95 ± 0.11	1.45 ± 0.09/1.81 ± 0.09	1.42 ± 0.04/2.02 ± 0.11
Heart	0.49 ± 0.03/0.50 ± 0.03	0.47 ± 0.06/0.49 ± 0.04	0.47 ± 0.05/0.48 ± 0.05	0.46 ± 0.06/0.55 ± 0.06
Lymph node	0.02 ± 0.00/0.03 ± 0.01	0.02 ± 0.01/0.04 ± 0.02	0.03 ± 0.01/0.03 ± 0.01	0.03 ± 0.01/0.02 ± 0.01
Spleen	0.29 ± 0.05/0.40 ± 0.04	0.28 ± 0.05/0.36 ± 0.03	0.29 ± 0.04/0.43 ± 0.09	0.27 ± 0.05/0.38 ± 0.03
Thymus	0.13 ± 0.04/0.20 ± 0.07	0.15 ± 0.02/0.28 ± 0.08	0.15 ± 0.03/0.24 ± 0.05	0.13 ± 0.03/0.21 ± 0.03
Liver	4.39 ± 0.26/4.25 ± 0.23	4.24 ± 0.22/4.32 ± 0.18	4.51 ± 0.35/4.48 ± 0.29	4.15 ± 0.09/4.53 ± 0.20
Pancreas	0.52 ± 0.06/0.53 ± 0.05	0.54 ± 0.08/0.53 ± 0.05	0.53 ± 0.08/0.52 ± 0.03	0.55 ± 0.08/0.57 ± 0.03
Lung	0.51 ± 0.02/0.64 ± 0.03	0.52 ± 0.04/0.67 ± 0.05	0.52 ± 0.04/0.65 ± 0.03	0.51 ± 0.03/0.67 ± 0.03
Kidney	0.80 ± 0.08/0.67 ± 0.05	0.77 ± 0.09/0.64 ± 0.05	0.83 ± 0.15/0.63 ± 0.04	0.75 ± 0.08/0.70 ± 0.03
Adrenal gland	0.02 ± 0.01/0.02 ± 0.01	0.01 ± 0.01/0.02 ± 0.01	0.02 ± 0.01/0.02 ± 0.01	0.02 ± 0.01/0.02 ± 0.01
Testis	0.32 ± 0.02/—	0.31 ± 0.05/—	0.31 ± 0.06/—	0.32 ± 0.04/—
Epididymis	0.13 ± 0.02/—	0.13 ± 0.02/—	0.12 ± 0.01/—	0.12 ± 0.01/—
Ovary	—/0.13 ± 0.06	—/0.12 ± 0.04	—/0.09 ± 0.01	—/0.09 ± 0.04
Uterus	—/0.59 ± 0.22	—/0.56 ± 0.23	—/0.57 ± 0.31	—/0.76 ± 0.24

Mice (*n* = 5 per group each) were orally administered distilled water (Control) or fGT. The relative organ weight to the body weight (%) is expressed as mean ± standard deviation (SD).

**Table 3:** Histopathological abnormalities after a single oral dose of fGT.

	Control (male, female)	fGT (male, female)		
		2.0 g/kg	1.0 g/kg	0.5 g/kg
LN: LC hyperplasia +1	1/10 (1/5, 0/5)	1/10 (1/5, 0/5)	2/10 (1/5, 1/5)	1/10 (1/5, 0/5)
Spleen: LC decrease +1	3/10 (1/5, 2/5)	1/10 (1/5, 0/5)	1/10 (0/5, 1/5)	2/10 (1/5, 1/5)
Liver: IF infiltration +1	0/10 (0/5, 0/5)	0/10 (0/5, 0/5)	1/10 (0/5, 1/5)	0/10 (0/5, 0/5)
Lung: congestion +1	3/10 (2/5, 1/5)	2/10 (1/5, 1/5)	2/10 (1/5, 1/5)	2/10 (1/5, 1/5)

Mice (*n* = 5 per group each) were orally administered distilled water (Control) or fGT. The score +1 means slight abnormal changes. Mice showing the abnormalities are listed as a number per total number. LN = lymph node; LC = lymphoid cell; IF = inflammatory cell.

**Table 4:** Hematological changes after a repeated oral dose of fGT for 13 weeks.

	Control (male, female)	fGT (male, female)		
		2.0 g/kg	1.0 g/kg	0.5 g/kg
Erythrocyte (M/*μ*L)	7.63 ± 0.23/6.86 ± 0.58	7.95 ± 0.62/6.58 ± 0.44	7.66 ± 0.57/6.92 ± 0.79	7.72 ± 0.29/6.75 ± 0.92
Hematocrit (%)	64.94 ± 2.23/62.32 ± 3.81	67.28 ± 1.89/59.82 ± 2.91	62.34 ± 4.55/62.38 ± 5.19	64.32 ± 1.58/62.52 ± 7.04
Hemoglobin (g/dL)	13.90 ± 0.50/13.78 ± 0.49	14.35 ± 0.26/13.38 ± 0.70	13.62 ± 1.02/13.34 ± 1.07	14.04 ± 0.55/13.30 ± 1.41
MCV (fL)	85.12 ± 1.30/90.96 ± 3.58	82.38 ± 2.94/91.00 ± 3.12	81.42 ± 2.05/90.42 ± 2.89	83.40 ± 1.36/92.82 ± 2.83
MCH (pg)	18.22 ± 0.28/20.20 ± 1.66	17.58 ± 0.74/19.76 ± 0.90	17.78 ± 0.38/19.34 ± 0.76	18.20 ± 0.45/19.76 ± 0.81
MCHC (g/dL)	21.62 ± 0.44/22.18 ± 1.71	21.33 ± 0.46/21.70 ± 0.41	21.84 ± 0.26/21.40 ± 0.25	21.84 ± 0.42/21.32 ± 0.25
Leukocytes (K/*μ*L)	8.75 ± 1.80/5.84 ± 2.26	8.42 ± 1.99/7.27 ± 2.84	9.45 ± 1.93/5.69 ± 1.16	7.43 ± 0.67/5.61 ± 2.32
Neutrophils (%)	15.34 ± 1.71/14.08 ± 3.45	18.10 ± 7.41/17.82 ± 2.99	18.10 ± 5.06/17.60 ± 4.43	18.58 ± 7.75/15.46 ± 12.23
Eosinophils (%)	1.91 ± 0.96/2.27 ± 0.46	0.93 ± 0.27/1.94 ± 0.59	1.95 ± 0.64/2.33 ± 0.49	1.88 ± 0.63/1.78 ± 0.81
Basophils (%)	3.01 ± 0.27/3.37 ± 0.90	3.16 ± 0.35/2.85 ± 0.41	2.85 ± 0.62/3.03 ± 1.33	2.17 ± 0.12^*∗*^/3.18 ± 1.18
Lymphocytes (%)	78.84 ± 2.21/79.40 ± 2.94	77.25 ± 7.74/77.02 ± 2.30	76.24 ± 4.74/75.86 ± 5.31	76.92 ± 7.93/78.38 ± 11.78
Monocytes (%)	0.95 ± 0.66/0.89 ± 0.54	0.60 ± 0.21/0.36 ± 0.34	0.86 ± 0.43/1.19 ± 1.50	0.45 ± 0.31/1.22 ± 1.02
Platelets (K/nL)	0.84 ± 0.18/0.74 ± 0.17	0.98 ± 0.08/0.86 ± 0.12	0.99 ± 0.10/0.86 ± 0.15	0.97 ± 0.14/0.59 ± 0.27

Rats (*n* = 5 per group each) were orally administered distilled water (Control) or fGT. The hematological items are expressed as mean ± SD. ^*∗*^*P* < 0.05 versus the control group by the Scheffe test. MCV = mean corpuscular volume; MCH = MC hemoglobin; MCHC = MCH concentration.

**Table 5:** Serum biochemical changes after a repeated oral dose of fGT for 13 weeks.

	Control (male/female)	fGT (male/female)		
		2.0 g/kg	1.0 g/kg	0.5 g/kg
ALP (IU/dL)	32.42 ± 13.85/20.18 ± 7.41	28.90 ± 6.16/22.02 ± 8.27	31.62 ± 11.75/21.84 ± 9.09	28.40 ± 4.37/18.34 ± 6.36
AST (IU/dL)	12.48 ± 4.10/10.70 ± 2.54	10.86 ± 2.01/9.86 ± 0.73	10.84 ± 1.25/10.74 ± 3.59	12.38 ± 2.62/9.04 ± 1.10
ALT (IU/L)	32.80 ± 8.17/27.80 ± 5.50	27.60 ± 4.72/24.40 ± 4.10	25.40 ± 3.21/36.00 ± 29.07	26.40 ± 4.45/25.80 ± 8.73
LDH (IU/dL)	90.00 ± 0.00/86.94 ± 6.84	90.00 ± 0.00/90.00 ± 0.00	86.60 ± 7.60/88.58 ± 3.18	90.00 ± 0.00/87.32 ± 5.99
BUN (mg/dL)	14.60 ± 1.47/13.50 ± 2.38	14.54 ± 2.03/14.88 ± 3.15	13.82 ± 1.76/13.78 ± 1.11	13.80 ± 0.82/14.90 ± 2.39
Glucose (mg/dL)	142.80 ± 27.94/137.00 ± 23.00	148.20 ± 22.58/128.20 ± 21.23	155.00 ± 10.32/145.80 ± 26.83	126.60 ± 13.79/133.20 ± 26.29
Total cholesterol (mg/dL)	67.80 ± 12.72/85.60 ± 12.26	66.60 ± 16.67/80.20 ± 12.26	61.00 ± 6.56/80.66 ± 42.10	57.20 ± 7.19/77.60 ± 15.42
Triglyceride (mg/dL)	80.20 ± 21.83/76.60 ± 10.14	110.20 ± 46.56/78.80 ± 50.30	94.20 ± 18.82/79.00 ± 40.68	91.00 ± 25.56/81.40 ± 43.00
CPK (IU/dL)	51.18 ± 21.02/29.48 ± 11.98	39.16 ± 16.76/26.78 ± 6.62	36.72 ± 13.05/23.10 ± 8.59	42.08 ± 16.89/22.00 ± 6.25
Creatinine (mg/dL)	0.30 ± 0.00/0.38 ± 0.11	0.32 ± 0.04/0.36 ± 0.05	0.30 ± 0.00/0.32 ± 0.04	0.28 ± 0.04/0.28 ± 0.04
Total bilirubin (mg/dL)	0.28 ± 0.08/0.38 ± 0.35	0.20 ± 0.00/0.34 ± 0.11	0.24 ± 0.05/0.30 ± 0.00	0.14 ± 0.05^#^/0.42 ± 0.18
Total protein (g/dL)	6.18 ± 0.23/6.90 ± 0.71	6.32 ± 0.41/6.46 ± 0.66	6.18 ± 0.33/7.26 ± 0.48	6.08 ± 0.26/6.32 ± 0.83
Albumin (g/dL)	3.80 ± 0.23/4.74 ± 0.53	4.00 ± 0.19/4.40 ± 0.58	3.96 ± 0.17/5.18 ± 0.31	3.66 ± 0.15/4.22 ± 0.53
Globulin (g/dL)	2.38 ± 0.26/2.16 ± 0.31	2.32 ± 0.34/2.06 ± 0.11	2.22 ± 0.29/2.08 ± 0.37	2.42 ± 0.30/2.10 ± 0.44
Albumin/Globulin ratio	1.62 ± 0.25/2.22 ± 0.31	1.75 ± 0.22/2.13 ± 0.23	1.81 ± 0.28/2.55 ± 0.43	1.54 ± 0.24/2.06 ± 0.36
Phosphorus (mg/dL)	9.12 ± 1.61/7.12 ± 1.54	9.20 ± 1.30/7.38 ± 0.61	9.40 ± 1.20/7.00 ± 0.53	9.12 ± 1.05/7.68 ± 0.96
Calcium (mg/dL)	10.48 ± 0.73/10.58 ± 1.02	10.56 ± 0.85/10.68 ± 0.28	10.56 ± 0.80/11.26 ± 0.48	9.98 ± 0.34/11.08 ± 0.40
Sodium (mmol/dL)	14.06 ± 0.26/14.26 ± 0.17	13.98 ± 0.21/14.20 ± 0.07	14.22 ± 0.13/14.02 ± 0.08^*∗*^	14.34 ± 0.13/14.08 ± 0.11
Potassium (mmol/L)	5.68 ± 1.67/4.28 ± 0.97	6.50 ± 2.06/4.58 ± 0.88	5.58 ± 1.26/4.36 ± 1.10	4.60 ± 1.02/4.30 ± 0.78
Chloride (mmol/dL)	10.46 ± 0.21/10.74 ± 0.18	10.48 ± 0.16/10.54 ± 0.11	10.58 ± 0.0.29/10.68 ± 0.26	10.90 ± 0.14^*∗*^/10.66 ± 0.29

Rats (*n* = 5 per group each) were orally administered distilled water (Control) or fGT. The serum biochemical items are expressed as mean ± SD. ^*∗*^*P* < 0.05 versus the control group by the Scheffe test; ^#^*P* < 0.05 versus the control group by the MW test. ALP = alkaline phosphatase; AST = aspartate aminotransferase; ALT =  alanine aminotransferase; BUN = blood urea nitrogen; CPK = creatine phosphokinase; LDH = lactate dehydrogenase.

**Table 6:** Postmortem abnormalities after a repeated dose of fGT for 13 weeks.

	Control (male/female)	fGT (male/female)		
		2.0 g/kg	1.0 g/kg	0.5 g/kg
LN: hypertrophy +1	5/10 (2/5, 3/5)	7/10 (3/5, 4/5)	5/10 (2/5, 3/5)	5/10 (2/5, 3/5)
Lung: congestion +1	4/10 (2/5, 2/5)	4/10 (2/5, 2/5)	3/10 (1/5, 2/5)	4/10 (2/5, 2/5)

Rats (*n* = 5 per group each) were orally administered distilled water (Control) or fGT. The score +1 means slight abnormal changes. Rats showing the abnormalities are listed as a number per total number. LN = lymph node.

**Table 7:** Relative organ weights after a repeated dose of fGT for 13 weeks.

	Control (male/female)	fGT (male/female)		
		2.0 g/kg	1.0 g/kg	0.5 g/kg
Brain	0.40 ± 0.05/0.66 ± 0.04	0.41 ± 0.03/0.63 ± 0.06	0.39 ± 0.05/0.64 ± 0.04	0.41 ± 0.04/0.67 ± 0.04
Heart	0.27 ± 0.02/0.30 ± 0.02	0.27 ± 0.02/0.29 ± 0.02	0.28 ± 0.03/0.29 ± 0.03	0.28 ± 0.02/0.30 ± 0.03
Lymph node	0.01 ± 0.00/0.02 ± 0.01	0.01 ± 0.00/0.01 ± 0.00	0.01 ± 0.00/0.02 ± 0.01	0.01 ± 0.00/0.01 ± 0.01
Spleen	0.14 ± 0.01/0.19 ± 0.02	0.14 ± 0.02/0.18 ± 0.02	0.14 ± 0.01/0.18 ± 0.03	0.14 ± 0.01/0.18 ± 0.04
Thymus	0.06 ± 0.01/0.09 ± 0.01	0.08 ± 0.02/0.09 ± 0.02	0.07 ± 0.01/0.09 ± 0.02	0.06 ± 0.01/0.10 ± 0.01
Liver	2.42 ± 0.22/2.47 ± 0.19	2.60 ± 0.39/2.36 ± 0.05	2.53 ± 0.20/2.51 ± 0.34	2.39 ± 0.16/2.52 ± 0.32
Pancreas	0.19 ± 0.03/0.22 ± 0.02	0.22 ± 0.02/0.24 ± 0.06	0.18 ± 0.05/0.22 ± 0.02	0.19 ± 0.03/0.25 ± 0.05
Lung	0.27 ± 0.02/0.37 ± 0.02	0.27 ± 0.03/0.36 ± 0.03	0.28 ± 0.02/0.36 ± 0.03	0.28 ± 0.02/0.38 ± 0.03
Kidney	0.30 ± 0.03/0.30 ± 0.02	0.31 ± 0.03/0.28 ± 0.02	0.30 ± 0.05/0.28 ± 0.03	0.28 ± 0.01/0.30 ± 0.04
Adrenal gland	0.01 ± 0.00/0.02 ± 0.00	0.01 ± 0.00/0.01 ± 0.00	0.01 ± 0.00/0.02 ± 0.00	0.01 ± 0.00/0.02 ± 0.00
Urinary bladder	0.03 ± 0.01/0.03 ± 0.01	0.03 ± 0.01/0.03 ± 0.01	0.03 ± 0.01/0.03 ± 0.01	0.03 ± 0.00/0.03 ± 0.01
Testis	0.33 ± 0.04/—	0.33 ± 0.03/—	0.31 ± 0.04/—	0.40 ± 0.05/—
Epididymis	0.14 ± 0.01/—	0.14 ± 0.01/—	0.13 ± 0.01/—	0.15 ± 0.02/—
Prostate	0.18 ± 0.03/—	0.18 ± 0.03/—	0.19 ± 0.01/—	0.21 ± 0.05/—
Ovary	—/0.03 ± 0.01	—/0.03 ± 0.00	—/0.03 ± 0.01	—/0.04 ± 0.01
Uterus	—/0.23 ± 0.04	—/0.24 ± 0.10	—/0.27 ± 0.08	—/0.26 ± 0.09

Rats (*n* = 5 per group each) were orally administered distilled water (Control) or fGT. The relative organ weight to the body weight (%) is expressed as mean ± SD.

**Table 8:** Histopathological abnormalities after a repeated oral dose of fGT for 13 weeks.

	Control (male/female)	fGT (male/female)		
		2.0 g/kg	1.0 g/kg	0.5 g/kg
LN: LC hyperplasia +1	5/10 (2/5, 3/5)	7/10 (3/5, 4/5)	5/10 (2/5, 3/5)	5/10 (2/5, 3/5)
Spleen: LC decrease +1	2/10 (1/5, 1/5)	2/10 (1/5, 1/5)	3/10 (1/5, 2/5)	0/10 (0/5, 0/5)
Thymus: congestion +1	1/10 (1/5, 0/5)	1/10 (1/5, 0/5)	0/10 (0/5, 0/5)	0/10 (0/5, 0/5)
Liver: IF infiltration +1	4/10 (1/5, 3/5)	2/10 (1/5, 1/5)	3/10 (1/5, 2/5)	4/10 (1/5, 3/5)
Lung: congestion +1	4/10 (2/5, 2/5)	4/10 (2/5, 2/5)	3/10 (1/5, 2/5)	4/10 (2/5, 2/5)
Adrenal gland: congestion +1	1/10 (0/5, 1/5)	1/10 (0/5, 1/5)	0/10 (0/5, 0/5)	0/10 (0/5, 0/5)
Prostate				
IF infiltration +1	2/5 (2/5, —)	1/5 (1/5, —)	2/5 (2/5, —)	1/5 (1/5, —)
Tubular atrophy +1	1/5 (1/5, —)	1/5 (1/5, —)	1/5 (1/5, —)	1/5 (1/5, —)
Pylorus: cyst +1	0/10 (0/5, 0/5)	0/10 (0/5, 0/5)	1/10 (0/5, 1/5)	0/10 (0/5, 0/5)

Rats (*n* = 5 per group each) were orally administered distilled water (Control) or fGT. The score +1 means slight abnormal changes. Rats showing the abnormalities are listed as a number per total number. LN = lymph nodes; LC = lymphoid cell; IF = inflammatory cell.

## Data Availability

The data used to support the findings of this study are available from the corresponding author upon request.

## Supplementary Materials

Supplementary Materials Absolute organ weights after a single oral dose of fGT and after the repeated oral dose for 13 weeks are presented in Tables S1 and S2, respectively. Body weight changes after the single oral dose are presented in Figure S1. Histopathological changes after the single oral dose and after the repeated oral dose for 13 weeks are presented in Figures S2 and S3, respectively.

## References

[1] McKay D. L., Blumberg J. B. (2002). The role of tea in human health: an update. *Journal of the American College of Nutrition*.

[2] Namita P., Mukesh R., Vijay K. J. (2012). Camellia sinensis (green tea): a review. *Global Journal of Pharmacology*.

[3] Sharangi A. B. (2009). Medicinal and therapeutic potentialities of tea (*Camellia sinensis* L.)—a review. *Food Research International*.

[4] Seeram N. P., Henning S. M., Niu Y., Lee R., Scheuller H. S., Heber D. (2006). Catechin and caffeine content of green tea dietary supplements and correlation with antioxidant capacity. *Journal of Agricultural and Food Chemistry*.

[5] Syu K. Y., Lin C. L., Huang H. C., Lin J. K. (2008). Determination of theanine, GABA, and other amino acids in green, oolong, black, and Pu-erh teas with dabsylation and high-performance liquid chromatography. *Journal of Agricultural and Food Chemistry*.

[6] Lin J.-K., Lin C.-L., Liang Y.-C., Lin-Shiau S.-Y., Juan I.-M. (1998). Survey of catechins, gallic acid, and methylxanthines in green, oolong, pu-erh, and black teas. *Journal of Agricultural and Food Chemistry*.

[7] Hubbe M., Joubert E. (2000). *Hydrogen Donating Ability of Honeybush Tea (Cyclopia intermedia) as a Measure of Antioxidant Activity*.

[8] Wang Z. M., Zhou B., Wang Y. S. (2011). Black and green tea consumption and the risk of coronary artery disease: a meta-analysis. *American Journal of Clinical Nutrition*.

[9] Montague J. A., Butler L. M., Wu A. H. (2012). Green and black tea intake in relation to prostate cancer risk among Singapore Chinese. *Cancer Causes & Control*.

[10] Zheng J. S., Yang J., Fu Y. Q., Huang T., Huang Y. J., Li D. (2013). Effects of green tea, black tea, and coffee consumption on the risk of esophageal cancer: a systematic review and meta-analysis of observational studies. *Nutrition and Cancer*.

[11] Hu J., Webster D., Cao J., Shao A. (2018). The safety of green tea and green tea extract consumption in adults—results of a systematic review. *Regulatory Toxicology and Pharmacology*.

[12] Teschke R., Zhang L., Melzer L., Schulze J., Eickhoff A. (2014). Green tea extract and the risk of drug-induced liver injury. *Expert Opinion on Drug Metabolism & Toxicology*.

[13] Lopez T. E., Pham H. M., Barbour J. (2016). The impact of green tea polyphenols on development and reproduction in *Drosophila melanogaster*. *Journal of Functional Foods*.

[14] Lin Y. S., Tsai Y. J., Tsay J. S., Lin J. K. (2003). Factors affecting the levels of tea polyphenols and caffeine in tea leaves. *Journal of Agricultural and Food Chemistry*.

[15] Jung Y. M., Lee S. H., Lee D. S. (2011). Fermented garlic protects diabetic, obese mice when fed a high-fat diet by antioxidant effects. *Nutrition Research*.

[16] Kang S. J., Lee J. E., Lee E. K. (2014). Fermentation with *Aquilariae lignum* enhances the anti-diabetic activity of green tea in type II diabetic db/db mouse. *Nutrients*.

[17] Kim C. M., Yi S. J., Cho I. J., Ku S. K. (2013). Red-koji fermented red ginseng ameliorates high fat diet-induced metabolic disorders in mice. *Nutrients*.

[18] Takagi K., Kimura M., Harada M., Otsuka Y. (1982). *Pharmacology of Medicinal Herbs in East Asia*.

[19] Nguyen T. T. T., Nguyen V. D. (2014). Biodiversity of major bacterial groups in association with agarwood (*Aquilaria crassna*) in Khanh Hoa province, Vietnam. *Journal of Vietnamese Environment*.

[20] Yin J. (1995). *Modern Research and Clinical Applications of Chinese Materia Medica*.

[21] Zhang Y., Wang W., Zhang J. (2004). Effects of novel anxiolytic 4-butyl-alpha-agarofuran on levels of monoamine neurotransmitters in rats. *European Journal of Pharmacology*.

[22] Kim Y., Lee E., Lee Y. (1997). Effect of the aqueous extract of *Aquilaria agallocha* stems on the immediate hypersensitivity reactions. *Journal of Ethnopharmacology*.

[23] Okugawa H., Ueda R., Matsumoto K., Kawanishi K., Kato A. (1996). Effect of jinkoh-eremol and agarospirol from agarwood on the central nervous system in mice. *Planta Medica*.

[24] Wang S. L., Hwang T. L., Chung M. I. (2015). New flavones, a 2-(2-phenylethyl)-4H-chromen-4-one derivative, and anti-inflammatory constituents from the stem barks of *Aquilaria sinensis*. *Molecules*.

[25] Dahham S. S., Tabana Y. M., Iqbal M. A. (2015). The anticancer, antioxidant and antimicrobial properties of the sesquiterpene *β*-caryophyllene from the essential oil of *Aquilaria crassna*. *Molecules*.

[26] Lee J. E., Kang S. J., Choi S. H., Song C. H., Lee Y. J., Ku S. K. (2015). Fermentation of green tea with 2% *Aquilariae lignum* increases the anti-diabetic activity of green tea aqueous extracts in the high fat-fed mouse. *Nutrients*.

[27] OECD (2002). *Guideline for Testing of Chemicals: Acute Oral Toxicity–Acute Toxic Class Method*.

[28] KFDA (2013). *Testing Guidelines for Safety Evaluation of Drugs (Notification No. 2013-121, Issued by the Korea Food and Drug Administration on April 05, 2013)*.

[29] Dourish C. T. (1987). Effects of drugs on spontaneous motor activity. *Experimental Psychopharmacology*.

[30] Kim H. S., Park S. I., Choi S. H. (2015). Single oral dose toxicity test of blue honeysuckle concentrate in mice. *Toxicological Research*.

[31] Park M. Y., Choi H. Y., Kim J. D., Lee H. S., Ku S. K. (2010). 28 days repeated oral dose toxicity test of aqueous extracts of mahwangyounpae-tang, a polyherbal formula. *Food and Chemical Toxicology*.

[32] Wolford S. T., Schroer R. A., Gohs F. X. (1986). Reference range data base for serum chemistry and hematology values in laboratory animals. *Journal of Toxicology and Environmental Health*.

[33] Sur T. K., Chatterjee S., Hazra A. K., Pradhan R., Chowdhury S. (2015). Acute and sub-chronic oral toxicity study of black tea in rodents. *Indian Journal of Pharmacology*.

[34] Hsu Y. W., Tsai C. F., Chen W. K., Huang C. F., Yen C. C. (2011). A subacute toxicity evaluation of green tea (*Camellia sinensis*) extract in mice. *Food and Chemical Toxicology*.

[35] Isbrucker R. A., Edwards J. A., Wolz E., Davidovich A., Bausch J. (2006). Safety studies on epigallocatechin gallate (EGCG) preparations. Part 2: dermal, acute and short-term toxicity studies. *Food and Chemical Toxicology*.

[36] Lambert J. D., Kennett M. J., Sang S., Reuhl K. R., Ju J., Yang C. S. (2010). Hepatotoxicity of high oral dose (-)-epigallocatechin-3-gallate in mice. *Food and Chemical Toxicology*.

[37] Yang C. S., Zhang J., Zhang L., Huang J., Wang Y. (2016). Mechanisms of body weight reduction and metabolic syndrome alleviation by tea. *Molecular Nutrition & Food Research*.

[38] Zhang L., Han Y., Xu L. (2015). The effects of co-administration of butter on the absorption, metabolism and excretion of catechins in rats after oral administration of tea polyphenols. *Food & Function*.

[39] Chan P. C., Ramot Y., Malarkey D. E. (2010). Fourteen-week toxicity study of green tea extract in rats and mice. *Toxicologic Pathology*.

[40] Bhattacharyya A., Mandal D., Lahiry L., Sa G., Das T. (2004). Black tea protects immunocytes from tumor-induced apoptosis by changing Bcl-2/Bax ratio. *Cancer Letters*.

[41] Isbrucker R. A., Bausch J., Edwards J. A., Wolz E. (2006). Safety studies on epigallocatechin gallate (EGCG) preparations. Part 1: genotoxicity. *Food and Chemical Toxicology*.

[42] Takami S., Imai T., Hasumura M., Cho Y. M., Onose J., Hirose M. (2008). Evaluation of toxicity of green tea catechins with 90-day dietary administration to F344 rats. *Food and Chemical Toxicology*.

[43] Bedrood Z., Rameshrad M., Hosseinzadeh H. (2018). Toxicological effects of *Camellia sinensis* (green tea): a review. *Phytotherapy Research*.

[44] Nagai K., Oda A., Konishi H. (2015). Theanine prevents doxorubicin-induced acute hepatotoxicity by reducing intrinsic apoptotic response. *Food and Chemical Toxicology*.

[45] Lu X., Zhao Y., Sun Y., Yang S., Yang X. (2013). Characterisation of polysaccharides from green tea of Huangshan Maofeng with antioxidant and hepatoprotective effects. *Food Chemistry*.

[46] Feng L., Gao M. J., Hou R. Y. (2014). Determination of quality constituents in the young leaves of albino tea cultivars. *Food Chemistry*.

