# Supplementary material for: Acute and Subchronic Oral Toxicity of Fermented Green Tea with Aquilariae Lignum in Rodents
Source: Evid Based Complement Alternat Med. 2019 Sep 10;2019:8721858. doi: 10.1155/2019/8721858 (PMC6754909; doi:10.1155/2019/8721858)
Supplement: Supplementary Materials — Absolute organ weights after a single oral dose of fGT and after the repeated oral dose for 13 weeks are presented in Tables S1 and S2, respectively. Body weight changes after the single oral dose are presented in Figure S1. Histopathological changes after the single oral dose and after the repeated oral dose for 13 weeks are presented in Figures S2 and S3, respectively. [file 8721858.f1.docx]

Table S 1. Absolute organ weights after a single oral dose of fGT.

|  | Control (male/female) | fGT (male/female) | | |
| --- | --- | --- | --- | --- |
|  |  | 2.0 g/kg | 1.0 g/kg | 0.5 g/kg |
| Brain (g) | 0.46±0.01 / 0.47±0.02 | 0.46±0.02 / 0.47±0.01 | 0.47±0.02 / 0.46±0.02 | 0.45±0.03 / 0.48±0.01 |
| Heart (g) | 0.16±0.01 / 0.12±0.00 | 0.15±0.01 / 0.12±0.00 | 0.15±0.01 / 0.12±0.01 | 0.15±0.02 / 0.13±0.01 |
| Lymph node (mg) | 6.00±1.22 / 7.60±3.58 | 7.60±2.61 / 9.60±3.97 | 8.20±4.09 / 8.20±3.11 | 7.80±3.11 / 4.20±2.49 |
| Spleen (g) | 0.09±0.02 / 0.10±0.02 | 0.09±0.02 / 0.09±0.01 | 0.09±0.01 / 0.11±0.03 | 0.09±0.02 / 0.09±0.01 |
| Thymus (g) | 0.04±0.01 / 0.05±0.02 | 0.05±0.01 / 0.07±0.02 | 0.05±0.01 / 0.06±0.01 | 0.04±0.01 / 0.05±0.01 |
| Liver (g) | 1.40±0.12 / 1.05±0.09 | 1.34±0.14 / 1.05±0.03 | 1.46±0.14 / 1.14±0.08 | 1.33±0.08 / 1.07±0.09 |
| Pancreas (g) | 0.17±0.02 / 0.13±0.01 | 0.17±0.02 / 0.13±0.01 | 0.17±0.03 / 0.13±0.01 | 0.18±0.03 / 0.13±0.01 |
| Lung (g) | 0.16±0.00 / 0.16±0.01 | 0.17±0.01 / 0.16±0.01 | 0.17±0.01 / 0.17±0.01 | 0.16±0.01 / 0.16±0.01 |
| Kidney (g) | 0.25±0.02 / 0.16±0.01 | 0.24±0.02 / 0.16±0.02 | 0.27±0.05 / 0.16±0.01 | 0.24±0.03 / 0.16±0.01 |
| Adrenal gland (mg) | 5.40±2.88 / 4.20±1.64 | 4.00±1.58 / 5.00±2.00 | 6.20±1.64 / 5.00±2.12 | 7.80±3.70 / 3.80±2.39 |
| Testis (g) | 0.10±0.00 / - | 0.10±0.02 / - | 0.10±0.02 / - | 0.10±0.01 / - |
| Epididymis (g) | 0.04±0.01 / - | 0.04±0.01 / - | 0.04±0.00 / - | 0.04±0.00 / - |
| Ovary (g) | - / 0.03±0.02 | - / 0.03±0.01 | - / 0.02±0.00 | - / 0.02±0.01 |
| Uterus (g) | - / 0.15±0.05 | - / 0.14±0.05 | - / 0.15±0.08 | - / 0.18±0.06 |

Mice (n=5 per group each) were orally administered with distilled water (Control) or fGT. The organ weight was expressed as means ± standard deviation (SD).

Table S 2. Absolute organ weights after a repeated oral dose of fGT for 13 weeks

|  | Control (male/female) | fGT (male/female) | | |
| --- | --- | --- | --- | --- |
|  |  | 2.0 g/kg | 1.0 g/kg | 0.5 g/kg |
| Brain | 2.18±0.12 / 2.05±0.08 | 2.22±0.03 / 2.04±0.08 | 2.09±0.11 / 2.03±0.04 | 2.10±0.07 / 2.05±0.07 |
| Heart | 1.52±0.15 / 0.93±0.08 | 1.45±0.06 / 0.93±0.09 | 1.48±0.10 / 0.93±0.06 | 1.44±0.12 / 0.93±0.07 |
| Lymph node | 0.06±0.02 / 0.05±0.02 | 0.05±0.01 / 0.05±0.01 | 0.04±0.02 / 0.05±0.02 | 0.05±0.01 / 0.04±0.02 |
| Spleen | 0.75±0.12 / 0.58±0.06 | 0.78±0.15 / 0.59±0.06 | 0.75±0.04 / 0.55±0.07 | 0.74±0.08 / 0.56±0.09 |
| Thymus | 0.34±0.06 / 0.29±0.02 | 0.40±0.08 / 0.29±0.07 | 0.38±0.05 / 0.30±0.08 | 0.33±0.04 / 0.30±0.03 |
| Liver | 13.53±2.51 / 7.76±0.85 | 13.92±1.25 / 7.70±0.82 | 13.67±2.39 / 7.92±0.74 | 12.56±2.02 / 7.77±0.88 |
| Pancreas | 1.08±0.21 / 0.68±0.12 | 1.20±0.09 / 0.75±0.13 | 0.98±0.27 / 0.69±0.08 | 0.98±0.10 / 0.76±0.15 |
| Lung | 1.51±0.17 / 1.16±0.05 | 1.47±0.10 / 1.15±0.10 | 1.49±0.08 / 1.11±0.07 | 1.48±0.11 / 1.16±0.08 |
| Kidney | 1.69±0.31 / 0.92±0.09 | 1.66±0.17 / 0.91±0.10 | 1.59±0.21 / 0.90±0.07 | 1.49±0.15 / 0.91±0.08 |
| Adrenal gland | 0.03±0.01 / 0.05±0.01 | 0.04±0.01 / 0.05±0.01 | 0.03±0.00 / 0.05±0.01 | 0.04±0.00 / 0.05±0.01 |
| Urinary bladder | 0.17±0.06 / 0.09±0.02 | 0.16±0.02 / 0.10±0.01 | 0.17±0.03 / 0.10±0.01 | 0.18±0.03 / 0.09±0.02 |
| Testis | 1.82±0.15 / - | 1.75±0.07 / - | 1.64±0.19 / - | 1.84±0.25 / - |
| Epididymis | 0.76±0.07 / - | 0.77±0.05 / - | 0.71±0.07 / - | 0.78±0.06 / - |
| Prostate | 0.95±0.18 / - | 0.97±0.11 / - | 1.03±0.16 / - | 1.10±0.18 / - |
| Ovary | - / 0.11±0.03 | - / 0.10±0.02 | - / 0.10±0.03 | - / 0.11±0.03 |
| Uterus | - / 0.71±0.12 | - / 0.74±0.26 | - / 0.84±0.27 | - / 0.81±0.27 |

Rats (n=5 per group each) were orally administered with distilled water (Control) or fGT. Values were expressed as means ± SD.

Figure S1. Body weight changes after a single oral dose of fGT.


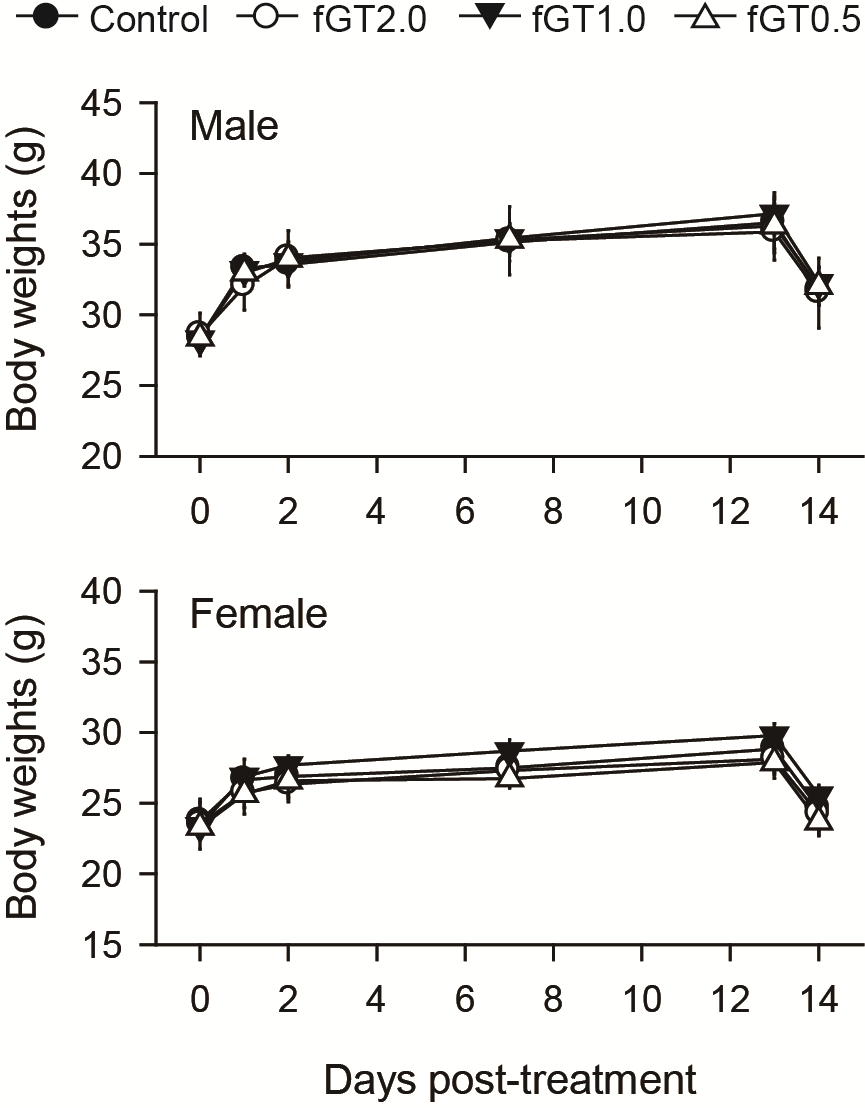


Body weights were expressed as means ± standard deviation (SD) of five mice in both genders.

Figure S2. Histopathological changes after a single oral dose of fGT


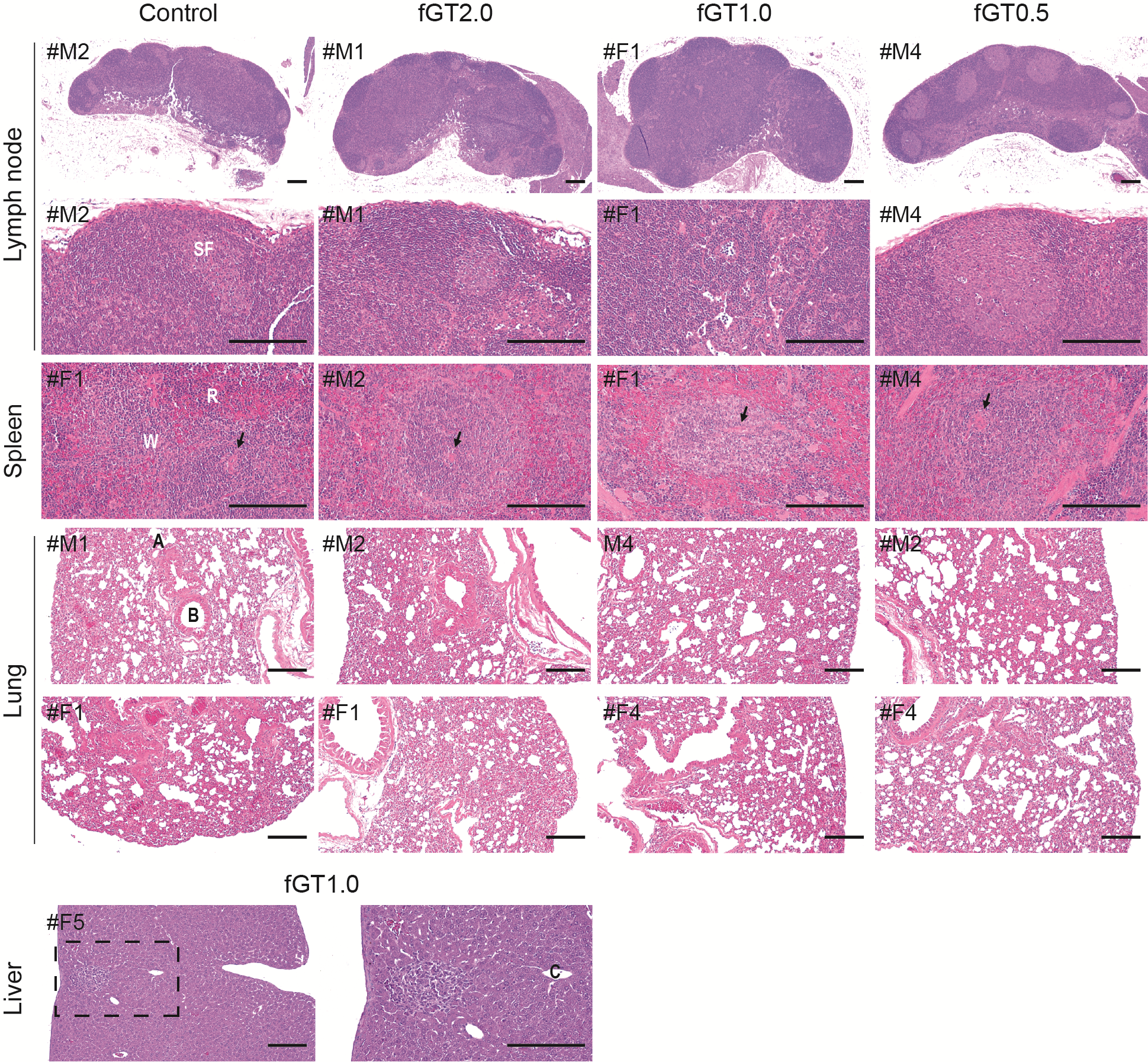


Representative pictures for histopathological changes in hematoxyline & eosin (H&E) stain are shown. Identification numbers of male (M) and female (F) mice in each group are indicated with #. Arrows in the spleen indicate central arterioles. Spotted area in the liver is magnified on the right. Scale bars = 200 µm. A = alveolar sac-respiratory bronchiole, B = primary bronchiole, C = central vein, R = red pulp, SF = secondary lymphatic follicle, W = white pulp.

Figure S3. Histopathological changes after a repeated oral dose of fGT for 13 weeks


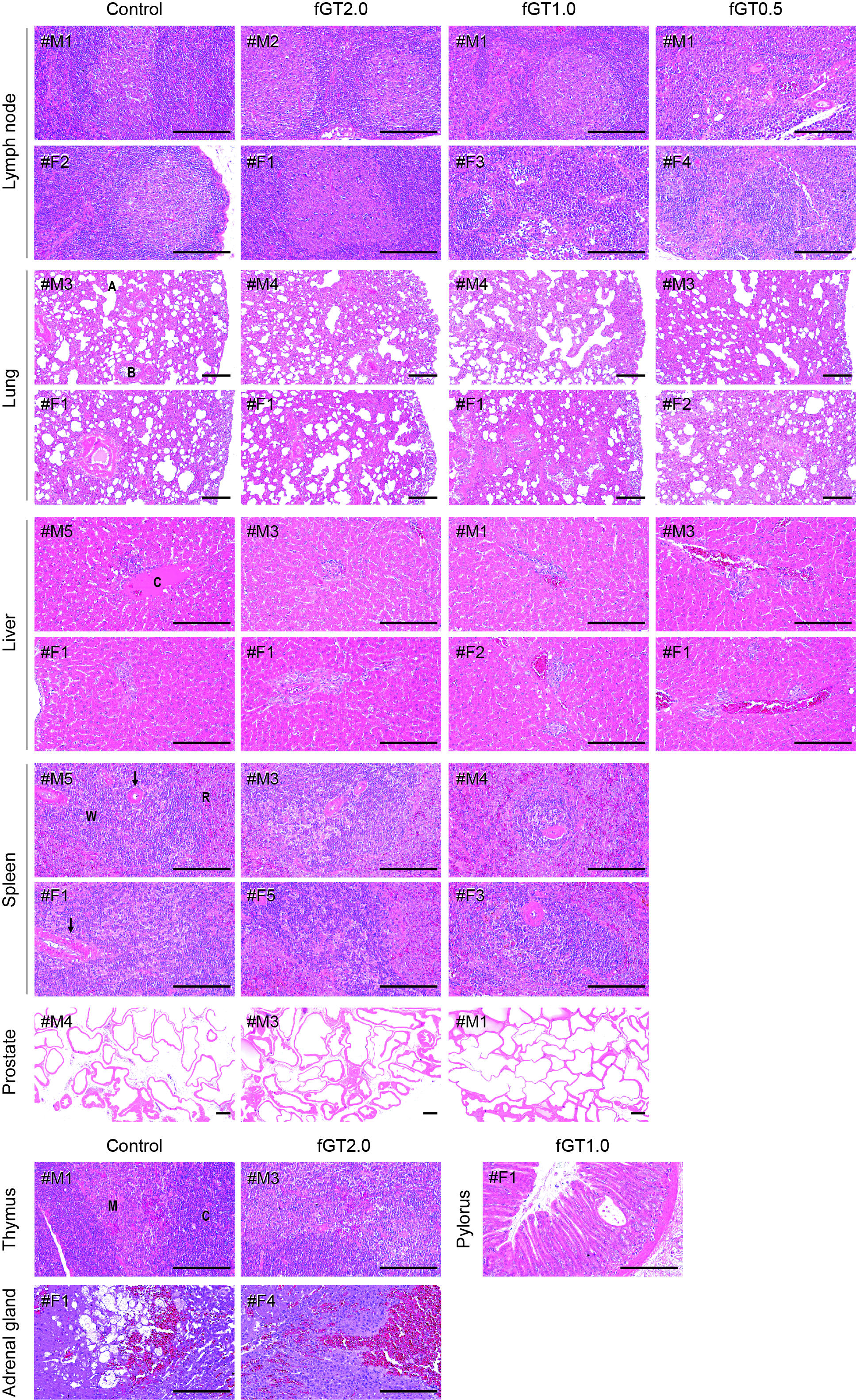


Representative pictures for histopathological changes in H&E stain are shown. Identification numbers of male (M) and female (F) rats in each group are indicated with #. Arrows in the spleen indicate central arterioles. Scale bars = 200 µm. A = alveolar sac-respiratory bronchiole, B = primary bronchiole, C = central vein, R = red pulp, SF = secondary lymphatic follicle, W = white pulp.
